# Physiology of invasive *Plasmodium* merozoites

**DOI:** 10.3389/fcimb.2026.1803461

**Published:** 2026-05-13

**Authors:** Mansoor A Siddiqui

**Affiliations:** Laboratory of Malaria and Vector Research, National Institute of Allergy and Infectious Diseases, National Institutes of Health, Rockville, MD, United States

**Keywords:** apicomplexan, gliding motility, malaria, merozoite, *Plasmodium*, protozoan parasites, rhoptry

## Abstract

Malaria, a life-threatening disease caused by *Plasmodium* parasites, relies on a specialized stage, the merozoite to invade red blood cells and multiply in the bloodstream. While much research has focused on the molecular interactions between merozoite and host cell surface, invasion is a complex process that also requires the parasite to move actively, sense its environment, and respond to signals such as changes in ions or contact with the red blood cell. These cues trigger precise biological responses, including the secretion of specialized organelles and activation of gliding motility, enabling the parasite to successfully enter the host cell. This review provides an overview of current understanding of merozoite physiology, highlighting how motility, signal sensing, and secretory organelle function are coordinated to drive invasion. By exploring these processes, we gain insights into potential strategies for malaria control and intervention.

## 1 Introduction

Malaria is a global public health concern, placing nearly half of the world’s population at risk of infection. In 2024, an estimated 282 million cases of malaria were recorded globally, resulting in approximately 610,000 deaths (World Health Organization, 2025). The re-emergence and persistence of malaria in regions affected by armed conflict, population displacement, and climate change highlight the uphill nature of malaria control efforts (World Health Organization, 2025). The limited availability and efficacy of currently available malaria vaccines and the emergence and spread of partial resistance to artemisinin-based antimalarial therapies substantially complicate global malaria elimination efforts. It is therefore essential to focus research efforts on understanding the biology of the malaria parasites.

*Plasmodium* species, the causative agents of malaria, are obligate intracellular parasites that spend major part of their complex life cycle within host cells. They transition only briefly into extracellular invasive stages, called zoites, which enable them to invade new host cells and continue the infection cycle. The most well-studied and clinically significant of these forms is the merozoite. The merozoite infects red blood cells in the bloodstream and multiplies asexually. Once internalized, the merozoite differentiates successively through ring stages into a trophozoite form that consumes red cell hemoglobin to undergo a unique form of asexual multiplication, referred as schizogony to produce 16–32 daughter merozoites (Rudlaff et al., 2020; Fréville et al., 2025). The daughter merozoites are released at the end of intra-erythrocytic multiplication through a well-orchestrated process known as egress and subsequently invade new red blood cells, thereby driving the exponential multiplication of the parasite in the bloodstream. The exponential multiplication of parasite within red blood cells is the basis of the all the pathology associated with the malaria (Miller et al., 2013).

**Figure 1:**
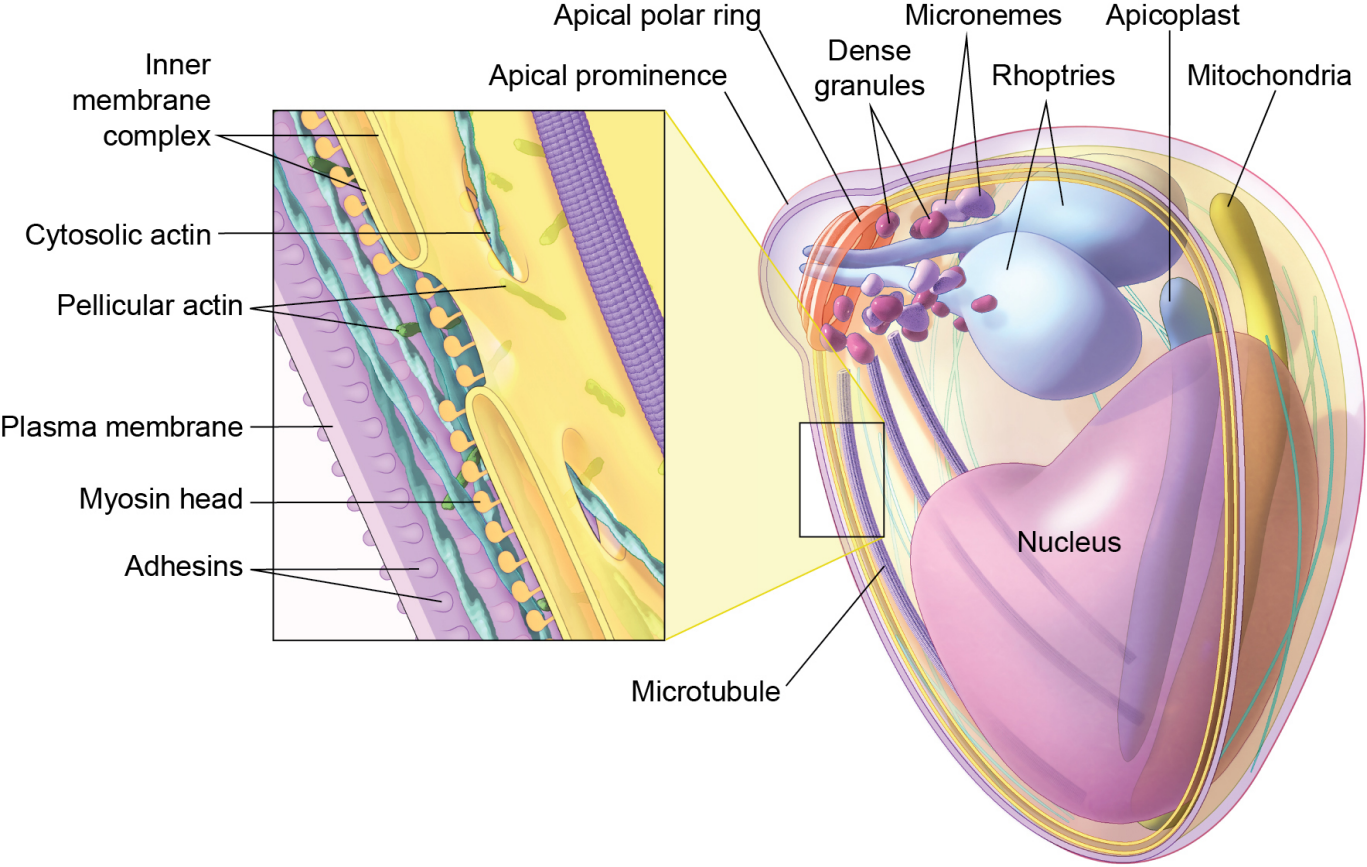
Cellular structure of a *Plasmodium* Merozoite. Note the broader end of the merozoite with apical organelles; Rhoptries, micronemes and dense granules. Note the apical prominence at the apical end of the merozoite and the localization of the apical polar rings. Also note the organelles and other cellular structures in the merozoite. Inset shows the organization of the components of the motility motor in the pellicular space. The pellicle is formed by the plasma membrane and inner-membrane complex. A section through the inner membrane complex (IMC) shows the fenestrations and its discontinuous nature. Myosin heads are tethered to the outer membrane of the IMC and are intimately associated with actin filaments in the pellicular space (Pellicular actin). Note the adhesins spanning the plasma membrane of the merozoite.

Merozoite is the smallest of all the life-cycle stages of the *Plasmodium* (*P. falciparum* merozoite is about ~1 µm in size) and notably the most abundant, due to exponential cyclic production during blood-stage infection. Nevertheless, the merozoite exhibit remarkable cellular and physiological adaptations to suit its role as an invasive cell (Hanssen et al., 2013; Aikawa et al., 1978; Singh et al., 2010). It is a polarized cell that has secretory organelles at the apical end and nucleus and other organelles at the distal end (**Figure 1**) (Hanssen et al., 2013; Aikawa et al., 1978). Extracellular merozoites sense and respond to multiple environmental cues, including physiological concentrations of K^+^ and Ca²^+^, as well as interactions with receptor molecules on the surface of host red blood cells (McCallum-Deighton and Holder, 1992; Singh et al., 2010). These stimuli induce the sequential release of apical organelles—micronemes and rhoptries and may also induce the merozoite motility (Singh et al., 2010; Siddiqui et al., 2020; Brochet et al., 2021). Thus, merozoites exhibit behavior reminiscent of excitable cells.

A conserved apicomplexan molecular machinery that consists of secretory apical organelles; the micronemes, rhoptries, and dense granules allows the merozoite to invade red blood cells (**Figure 1**) (Cowman et al., 2012). Microneme secretion is responsible for translocation of ligands or effector proteins to the merozoite surface (Healer et al., 2002; Bannister et al., 2003). The translocated ligands facilitate host cell recognition, stable attachment and force transmission for the merozoite motility, activities essential for invasion (Cowman et al., 2012). Rhoptries, two much larger club shaped secretory organelles in the merozoite mediate essential roles in facilitating host cell invasion and remodeling. Atypical nature of apical organelles defied conventional understanding of vesicle secretion from other models. Advances in *Plasmodium* experimental molecular genetics and functional genomics allowed identification and characterization of organelle-specific membrane fusion proteins, providing mechanistic insights into secretion of the micronemes and rhoptries. Cryogenic electron (Cryo-electron) microscopy has enabled the identification of an atypical supramolecular secretory complex—the rhoptry secretory apparatus (RSA)—in *Plasmodium* merozoites (Martinez et al., 2022). The RSA appears to function as a structural and regulatory hub that coordinates rhoptry docking and integrates signaling inputs into decisive secretion of rhoptries (Martinez et al., 2022; Aquilini et al., 2021). This review summarizes recent advances in the mechanisms of apical organelle secretion and highlights major unresolved questions, including how external signals are sensed, how secretion is precisely timed, and which molecular components directly mediate membrane fusion.

Red cell invasion depends on ability of merozoite to generate and translate the force into biomechanical events such as internalization through a molecular junction, also known as tight or moving junction. A functional actin–myosin motor in the merozoite has long been recognized as essential for host cell invasion (Baum et al., 2006; Green et al., 2008). Despite this evidence merozoites were assumed to lack the gliding motility (Baum et al., 2006), unlike sporozoites, ookinetes, or other apicomplexan zoites. However, video microscopy of larger and robust *Plasmodium Knowlesi* merozoites revealed bona fide gliding motility accompanied by a corkscrew motion during red blood cell invasion. The gliding motility is conserved among *Plasmodium* merozoites (Yahata et al., 2021). Demonstration of gliding motility also led to redefinition of merozoite morphology, identifying the broader end as the apical, invasive pole (**Figure 1**) (Yahata et al., 2021), contrary to assumptions that had persisted for more than five decades since the merozoite invasion was first recorded (Dvorak et al., 1975). In this review we aim to dissect the implications of the discovery of gliding motility for merozoite invasion and connect dots between the merozoite structure and physiology of gliding motility.

Extracellular, invasive merozoites represent the parasite’s most vulnerable stage, as they are directly exposed to the host immune system, making them a prime target for malaria vaccine development (Beeson et al., 2016). As a result, research has emphasized merozoite surface ligands that engage red blood cell receptors, while the cellular structure and physiology of the merozoite remained poorly understood. *Plasmodium* merozoites employ a repertoire of invasion ligands and can engage multiple erythrocyte receptors, resulting in substantial redundancy in invasion pathways, allowing merozoites to switch between alternative ligand–receptor interactions when one pathway is blocked (Duraisingh et al., 2003; Gaur et al., 2004) The biological redundancy in invasion pathways has significantly hindered efforts to develop an effective blood-stage malaria vaccine.

Invasive merozoites rely on tightly coordinated physiological processes—including the sensing of extracellular ionic and molecular cues, actomyosin-driven motility, and the regulated secretion of apical organelles—to achieve successful host cell invasion (Singh et al., 2010; Yahata et al., 2021). A deeper understanding of these fundamental mechanisms could reveal novel targets for chemotherapeutic or vaccine-based antimalarial interventions. Experimental investigations into merozoite biology are inherently challenging, given their fleeting extracellular viability diminutive size and fragility (Boyle et al., 2010). Advances in experimental genetics (Zhang et al., 2018) the discovery of potent egress inhibitors (Ressurreição et al., 2020), and major improvements in imaging technologies have collectively made the study of merozoite biology increasingly tractable. These innovations are driving novel insights that challenge long-standing paradigms, making this an exceptionally exciting era for foundational research on the merozoite, the tiny yet pivotal cell at the heart of the malaria parasite’s blood-stage lifecycle.

## 2 Physiology of apical organelle secretion

The complex life cycle of *Plasmodium* involves invasion of diverse host cell types in mosquito and vertebrate hosts. It requires a coordinated interplay between the secretion of apical organelles and motility. Secretion of apical organelles is tightly regulated and triggered in response to external signals sensed at the time of host cell invasion (Collins et al., 2013; Singh et al., 2010). Furthermore, secretion of apical organelles occurs hierarchically, micronemes are secreted before rhoptries, demonstrating the complex physiology that underpins host cell invasion (Singh et al., 2010).

The unusual and divergent nature of these secretory organelles made it challenging to interpret their mechanisms within the framework of well-characterized vesicle exocytosis pathways from the model eukaryotic unicellular organisms. SNARE proteins—key mediators of vesicle docking and fusion in canonical exocytosis, though conserved in apicomplexans, do not appear to play a central role in the regulated secretion of micronemes and rhoptries (Ayong et al., 2007; Aquilini et al., 2021). In recent years, however, advances in molecular genetics and functional genomics (Zhang et al., 2018) have led to the identification and characterization of novel molecular regulators that control apical organelle secretion in *Plasmodium* and *Toxoplasma* species, reshaping our understanding of this essential process.

Microneme secretion occurs in response to an increase in intracellular second-messenger abundance, primarily Ca^+2^ (Singh et al., 2010). This transient Ca^+2^ spike is attributed to its release from intracellular stores. The release of Ca^+2^ from the intracellular stores is mediated by Phospholipase-C (PLC) mediated signaling that is activated in the merozoites on exposure to low K^+^ environment (Bullen et al., 2016).

However, extracellular Ca^+2^ is essential for invasion (McCallum-Deighton and Holder, 1992), raising unresolved questions about the Ca^+2^ permeability of the merozoite plasma membrane and the presence of ion channels or transporters that facilitate influx of extracellular Ca²^+^ during motility and invasion. Furthermore, it has been demonstrated that merozoites are responsive to changes in the K^+^ concentration, experienced at the onset of egress exposing them from a high to a low concentration K^+^ milieu (Singh et al., 2010; Siddiqui et al., 2020). This shift appears to be an important physiological cue for microneme secretion (Singh et al., 2010; Siddiqui et al., 2020) yet the molecular basis of K^+^ sensing by the merozoites remains undefined. Once initiated, microneme secretion becomes constitutive, driving key physiological processes such as host cell recognition, adhesion and gliding motility, effectively preparing the merozoite for rapid entry into erythrocytes (Tagoe et al., 2021).

Emerging data suggest that microneme secretion sets the stage for rhoptry secretion by facilitating the surface translocation of several key microneme antigens, including EBA-175 (Erythrocyte Binding Antigen), AMA1 and CLAMP (Singh et al., 2010; Srinivasan et al., 2011; Valleau et al., 2023) Many of these molecules are implicated in mediating the signaling required for rhoptry secretion, and evidence supporting the role of AMA1 is particularly compelling. The Ca²^+^ surge that precedes the microneme secretion appears to be regulated by the activation of cyclic nucleotide signaling pathways, specifically cAMP and cGMP (Collins et al., 2013). The docking and fusion of micronemes with the merozoite plasma membrane, leading to secretion, occur in a lipid-dependent manner at the apical end of the merozoite. Early signaling events trigger changes in the lipid composition of the inner leaflet of the zoite plasma membrane (Bullen et al., 2016). These lipids are recognized by membrane fusion proteins on the microneme membrane, thereby providing the final signal required for microneme secretion already set in motion by Ca²^+^ surge. Microneme secretion is thus controlled by a complex signaling cascade, requiring the coordinated interplay of Ca²^+^ signaling and membrane lipid dynamics.

The secretion of rhoptries during red cell invasion marks a definitive step. Unlike the microneme secretion, where ligands are translocated to the surface of the merozoite to aid host cell recognition, binding and motility, the contents of the rhoptries (proteins and lipids) are injected directly into the host cell at the time of invasion (Martinez et al., 2022). Secreted proteins are required for the assembly of tight junction, the molecular nexus used by merozoite to push itself into the red blood cell. The lipids secreted from the rhoptries into the red blood cell contribute to the formation of the parasitophorous vacuole (PV) membrane (Ghosh et al., 2017; Mikkelsen et al., 1988). However, the nascent PV membrane is primarily formed by the host red blood cell membrane (Geoghegan et al., 2021). PV provides the intracellular niche in which the internalized merozoite undergoes further maturation and multiplication. Protein secretions from rhoptries are essential for remodeling of the host red cell. Most well-studied example is the injection of CLAG proteins into the host red blood cell, that constitute the ion channel PSAC (Plasmodium Surface Anion Channel) on the red blood cell membrane. PSAC plays a critical role in facilitating the nutrient uptake for intra erythrocytic parasite proliferation (Ito et al., 2017; Sherling et al., 2017).

### 2.1 Signaling and microneme secretion in the merozoites

Merozoites have a fleeting window of extracellular viability, quickly losing their invasive competence within minutes after egress. The process of red cell invasion, although complex is completed in less than a minute (Weiss et al., 2015). The merozoite signaling network is therefore wired for rapid signal detection and immediate execution. Ca^+2^ and cyclic nucleotides (cAMP and cGMP) constitute the second messengers in the merozoites (Dawn et al., 2014; Brochet et al., 2021; Collins et al., 2013). These second messengers activate downstream kinases that require Ca^+2^ or cyclic nucleotides for their activity. As expected, the physiology of invasion is regulated by phosphorylation of the merozoite proteome (Lasonder et al., 2015, 2012). The development of global phosphoproteomic approaches has further transformed the field by enabling systematic identification of phosphorylation events across the *Plasmodium* life cycle. Early methodological advances (Villén and Gygi, 2008) followed by malaria-specific applications allowed the mapping of phosphoproteomes in schizonts (Alam et al., 2015) and extracellular merozoites (Lasonder et al., 2015) and the interrogation of phosphorylation dynamics using genetic and chemical-genetic perturbations (Alam et al., 2015; Balestra et al., 2021). These studies identified extensive phosphorylation networks regulated by protein kinase A (PKA) and protein kinase G (PKG), thereby establishing Ca²^+^ and cyclic mononucleotides as central secondary messengers in the signaling pathways governing merozoite invasion (Singh et al., 2010; Dawn et al., 2014; Brochet et al., 2021).

Intracellular Ca²^+^ surges activate calcium-dependent protein kinases (CDPKs), which play critical roles in merozoite biology. Two essential CDPKs functioning in merozoites are *Pf*CDPK1 and *Pf*CDPK5 (Bansal et al., 2013; Dvorin et al., 2010). Activation of *Pf*CDPK5 is essential for the egress of merozoites and occurs downstream of *Pf*PKG. Conditional ablation of *Pf*CDPK5 in *Plasmodium* schizonts prevents egress despite an intact and functional PKG signaling pathway, indicating that *Pf*CDPK5 acts as an effector kinase required for merozoite release. However, *Pf*CDPK5-deficient merozoites do not exhibit impaired invasive competence; mechanically released parasites lacking *Pf*CDPK5 retain their ability to invade host cells (Dvorin et al., 2010). *Pf*CDPK5 is associated with the secretion of egress specific secretory organelles known as exonemes and is responsible for regulating their secretion or exocytosis (Absalon et al., 2018).

In contrast, both chemical and genetic inhibition of *Pf*CDPK1 significantly reduces the invasive capacity of merozoites. *Pf*CDPK1 is essential for the phosphorylation of components of the actomyosin motor complex (glideosome), which drives parasite motility (Green et al., 2008). Inhibition of *Pf*CDPK1 using purfalcamine—a potent inhibitor identified through high-throughput screening (Kato et al., 2008)—blocks the secretion of the microneme protein AMA1 (Bansal et al., 2013), a critical component required for tight junction formation during host cell invasion. *Pf*CDPK1 functions as a central signaling node regulating both the merozoite motor machinery (Jones et al., 2012; Kumar et al., 2017) and microneme secretion (Bansal et al., 2013). Furthermore, its localization to the merozoite membrane is controlled by dynamic protein S-palmitoylation in response to external ionic signals (Siddiqui et al., 2020), highlighting an additional layer of regulation that coordinates environmental sensing with invasion-related processes.

Despite these advances, current models of merozoite signaling remain incomplete. In particular, the mechanisms by which merozoites sense and interpret extracellular cues are poorly understood, precluding the definition of a coherent hierarchical ligand–receptor signaling framework for invasion. As in other excitable cells, the merozoite plasma membrane is likely to serve as a critical signaling interface, and recent studies have begun to uncover molecular components involved in membrane-proximal signal detection and transduction (Balestra et al., 2021; Brochet et al., 2021; Kuehnel et al., 2023). However, many critical components of the merozoite’s signal-sensing and transduction machinery are still poorly defined (Balestra et al., 2021).

Two closely spaced surges of intracellular Ca²^+^ have been reported during the transition from egress to invasion (Weiss et al., 2015; Hart et al., 2023). The first Ca²^+^ transient occurs immediately upon merozoite release following egress, as detected in Fluo-4 AM–loaded parasites, whereas a second, more pronounced Ca²^+^ surge is observed at the point of irreversible commitment to invasion, shortly after merozoite reorientation on the erythrocyte surface (Weiss et al., 2015; Hart et al., 2023). Quantitative fluorescence measurements indicate that these two Ca²^+^ elevations differ in magnitude, with the second surge being markedly stronger. However, how merozoites generate two temporally distinct and differentially regulated Ca²^+^ surges within a span of seconds remains mechanistically unresolved.

*Plasmodium* merozoites express an atypical guanylyl cyclase that is activated by as-yet poorly defined environmental cues (Kuehnel et al., 2023). Experimental evidence indicates that merozoites are sensitive to changes in extracellular potassium concentration and respond to this stimulus by elevating cytosolic Ca²^+^ levels (Singh et al., 2010), implicating ionic shifts encountered during egress or invasion as potential upstream triggers. Activation of the guanylyl cyclase at the zoite plasma membrane results in the production of cyclic guanosine monophosphate (cGMP), whose intracellular concentration is tightly controlled by the opposing activities of guanylyl cyclase and cGMP-specific phosphodiesterases. This dynamic regulation ensures precise temporal control of cGMP availability as a second messenger. cGMP signals exclusively through protein kinase G (PKG), which functions as its sole downstream effector (Balestra et al., 2021; Brochet et al., 2021). In *Plasmodium*, PKG activation is a key regulatory node controlling the mobilization of Ca²^+^ from intracellular stores (Balestra et al., 2021). The resulting Ca²^+^ surge is essential for the regulated secretion of egress- and invasion-associated effector proteins, including Subtilisin 1 and AMA1, from specialized secretory organelles such as exonemes and micronemes respectively (Collins et al., 2013).

Merozoites contain up to ~40 micronemes, which are elongated, long-necked, bottle-shaped organelles measuring approximately 160 nm in length and ~65 nm at their widest diameter (Bannister et al., 2003). Their external surfaces are decorated with bristle-like filaments, ~3–4 nm thick and ~25 nm long (Bannister et al., 2003). In *Plasmodium* merozoites, microneme secretion is controlled by the integration of Ca²^+^ signaling with phospholipid remodeling rather than by Ca²^+^ elevation alone. Activation of phospholipase C (PLC) results in the production of inositol trisphosphate (IP_3_) and diacylglycerol (DAG), with IP_3_ inducing Ca²^+^ release from intracellular stores (Singh et al., 2010; Bullen et al., 2016). In parallel, DAG is enzymatically converted into membrane-associated phosphatidic acid (PA), leading to localized PA enrichment at the parasite plasma membrane (Bullen et al., 2016). Phosphatidic acid (PA), with its relatively small head group, adopts a cone-shaped geometry that induces negative curvature when incorporated into lipid bilayers (Kooijman et al., 2005). This biophysical property makes PA a key facilitator of membrane remodeling events, including vesicle fusion. In the context of merozoites, PA is thought to play a critical role in the exocytosis of micronemes, where rapid membrane fusion is essential for the release of invasion effectors. By introducing negative curvature, PA may stabilize intermediate membrane structures, lower the energetic barrier for fusion, and ensure that secretion occurs efficiently and precisely at the apical tip (Zhukovsky et al., 2019). This lipid-mediated regulation highlights how biophysical properties of membranes are integrated within the signaling networks to orchestrate the tightly timed events required for successful red blood cell invasion. PA acts as a lipid docking signal for the Acylated Pleckstrin homology (APH) protein, that localizes to the surface of micronemes in *Plasmodium* (Bullen et al., 2016; Chaiyawong et al., 2022). Recruitment of APH to PA-enriched membrane at the apical end provides the additional spatial signal for microneme docking and fusion. Microneme secretion also requires the Ca²^+^ binding protein DOC2.1, that functions as a Ca²^+^ sensor and transduces the intracellular Ca²^+^ surge into a fusion-competent state (Farrell et al., 2012). Thus, in *Plasmodium*, DOC2.1 decodes the Ca²^+^ signal, while APH confers lipid-dependent spatial control, ensuring that microneme exocytosis occurs only at membranes enriched in PA.

Secretion of micronemes is necessary for the translocation of ligands such as MTRAP, AMA1 and EBAs to the merozoite surface necessary for motility, erythrocyte deformation and attachment and for successful internalization of merozoite. Functional characterization of membrane fusion proteins such as DOC2.1 and APH has provided important mechanistic insights into the process of microneme exocytosis, while also raising new questions. Evidence suggests that micronemes may exist as distinct subpopulations based on the specific antigens or ligands they contain. For instance, genetic ablation of APH in *Plasmodium yoelii* directly impairs the secretion of MTRAP but doesn’t have a direct effect on the release of AMA1 (Chaiyawong et al., 2022). This observation indicates that different microneme subsets may utilize distinct secretion pathways and exhibit varying dependencies on specific membrane fusion proteins.

### 2.2 Elusive nature of rhoptry secretion

Unlike the microneme secretion that follows a full vesicle fusion paradigm, triggered by a calcium spike, the rhoptry secretion cannot be induced by calcium ionophores or other small molecule inducers of microneme secretion and does not follow a full vesicle fusion paradigm. The rhoptry secretion is associated with the appearance of smooth, irregular vesicles in the host cell as the invading zoite advances, known as evacuoles (Håkansson et al., 2001; Riglar et al., 2011). Extracellular merozoites fixed with glutaraldehyde-tannic acid exhibited electron-dense multilamellar material, primarily membranous whorls attached to their surface, which is consistent with the release of rhoptry contents (Stewart et al., 1986). A form of aberrant rhoptry secretion has also been reported in the literature, where rhoptry markers are found to be secreted on the surface of extracellular merozoites or red blood cells. This peculiar phenomenon has been described as abortive merozoite invasion (Sterkers et al., 2007), merozoite fails to invade the red cell despite a rhoptry secretion. The physiology of rhoptry secretion remains poorly understood, particularly the mechanism by which rhoptry contents are injected into the host cell during invasion.

### 2.3 A conserved yet divergent secretion machinery regulates rhoptry discharge in merozoites

Advances in cryo-electron microscopy, coupled with improved sample preparation methods such as cryo-milling, have enabled high-resolution visualization of the ultrastructure of the much smaller merozoite stage of *Plasmodium* (Theveny et al., 2022; Martinez et al., 2022; Sun et al., 2024). These approaches revealed a rosette at the apical end of the merozoite, consistent with observation of similar structure in the zoites of *Toxoplasma* and *Cryptosporidium* (Martinez et al., 2022). The rosette comprises of intramembranous particles (IMPs) (Aquilini et al., 2021; Martinez et al., 2022). These IMPs are arranged with an eightfold rotational symmetry around a central axis, a structural feature conserved with the rosette observed in ciliates (Aquilini et al., 2021). Early electron microscopy studies identified structural parallels between apicomplexan rhoptries and defensive extrusive organelles such as trichocysts in *Paramecium* and mucocysts in *Tetrahymena*, present in free-living alveolates (Porchet and Torpier, 1977; Porchet-Hennere and Nicolas, 1983). Intramembranous rosette with eightfold rotational symmetry, marks a predetermined secretion site, where both organelles appear docked in a pre-fusion state, poised for stimulus-triggered discharge (Porchet and Torpier, 1977; Porchet-Hennere and Nicolas, 1983; Aquilini et al., 2021; Martinez et al., 2022).

A small vesicle positioned distal to the rhoptry tip was first described in freeze-fracture studies of *Toxoplasma* gondii zoites (Porchet and Torpier, 1977). Similarly, a distinct apical depression or pore—interpreted as the rhoptry discharge site—was observed in invading merozoites of *Plasmodium knowlesi* (Aikawa et al., 1981). However, contrast-enhanced cryo-electron tomography (Cryo-ET) has enabled visualization of sub-rosette structures that were not resolvable by classical freeze-fracture electron microscopy (Theveny et al., 2022; Aquilini et al., 2021; Martinez et al., 2022).

Molecular genetic studies in *Paramecium* identified key factors required for trichocyst discharge or secretion (Vayssié et al., 2000). The genes responsible, termed non-discharge (*nd*) genes, are essential for both rosette assembly and trichocyst exocytosis (Vayssié et al., 2000). These genes encode Nd proteins that localize to the plasma membrane in a rosette configuration, marking the predetermined site of discharge (Vayssié et al., 2000; Aquilini et al., 2021). Subsequent genome mining revealed homologous non-discharge (*nd*) and non-discharge partner (*ndP*) genes in *Plasmodium* and *Toxoplasma* (Aquilini et al., 2021; Martinez et al., 2022). Notably, *nd* and *ndP* genes are conserved across the alveolate superphylum, including members of Ciliata, Dinoflagellata, and Apicomplexa (Aquilini et al., 2021).

**Figure 2:**
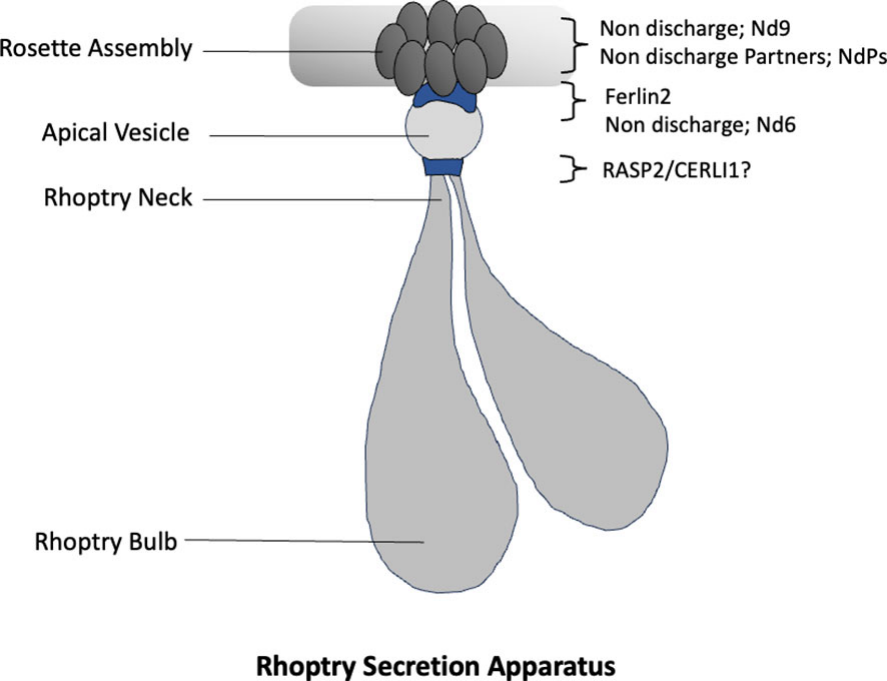
Outline of Rhoptry Secretion Apparatus. Organization of the components of Rhoptry secretion apparatus (RSA). A rosette of intramembranous particles is visible at the apex of merozoite plasma membrane, marking the site of secretion. The apical vesicle (AV) is positioned beneath this structure, serving as a docking platform for the rhoptries, which are seen anchored at their tips to the AV. Distinct membrane fusion proteins are observed at the interface between the rhoptry tips and the apical vesicle. Likewise, a complex supramolecular complex (RSA) present at the junction between the AV and the parasite plasma membrane, facilitates regulated exocytosis of rhoptries.

The intramembranous particles (IMPs) of the rosette in *Plasmodium* and other Apicomplexa are associated with a membranous spheroid, approximately 50 nm in diameter, that lies directly beneath them and is known as the apical vesicle (AV) (Aquilini et al., 2021; Martinez et al., 2022). The AV appears to be a unique adaptation for the parasitic mode of life of Apicomplexans because this structure is not present in free living ciliates such as *Paramaecium* but has been found associated with rosette in the *Toxoplasma* and *Cryptosporidium* zoites (Mageswaran et al., 2021). Apical vesicle together with the intra membranous rosette and associated molecular players constitutes what is now known as Rhoptry Secretion Apparatus or RSA. In majority of the imaged *Plasmodium* merozoites, AV appears to receive the two rhoptry necks (Martinez et al., 2022; Sun et al., 2024). The rhoptry tips dock at the AV and thus forming a conduit between the Rhoptry secretion complex and the rhoptries (**Figure 2**). In merozoites the apical vesicle (AV) disappears following rhoptry fusion, suggesting that its loss is concomitant with rhoptry secretion (Martinez et al., 2022). Moreover, the Cryo-ET has revealed changes in the conformations of the RSA following rhoptry secretion.

Conditional ablation of a non-discharge homolog gene, *nd*9 leads to disruption of rosette formation in *Plasmodium* merozoites and a defect in the secretion of rhoptries similar to what has been demonstrated in the *Toxoplasma* tachyzoite*s*. The *nd* and *ndP* genes are thus required for the assembly and function of the rhoptry secretion apparatus (RSA). Conditional knockout of *nd* genes results in a complete block of rhoptry secretion, while leaving microneme secretion, including the release of proteins such as AMA1, unaffected in *Plasmodium* merozoites (Aquilini et al., 2021; Martinez et al., 2022).

### 2.4 What triggers rhoptry secretion?

Rhoptry secretion occurs only after the merozoite has committed to the invasion. Commitment to invasion has a well-established molecular basis; high affinity interactions between the microneme ligands, EBAs and AMA1 translocated to the merozoite surface and their cognate receptors on the host red cell surface. Thus, physical contact with the host cell surface through these ligands appears to be necessary for triggering rhoptry secretion. Notably, two ligand-receptor pairs have been implicated in triggering rhoptry secretion in *Plasmodium* merozoites; EBA-175-Glycophorin A (Singh et al., 2010) and AMA1-RON2 (Srinivasan et al., 2011).

The AMA1-RON2 interaction requires the binding of RON2 protein (a rhoptry resident protein secreted at the time of invasion) to the binding groove in the AMA1 protein (Lamarque et al., 2011; Tyler and Boothroyd, 2011). This interaction is the molecular basis of the tight junction formation, can be inhibited either with anti AMA1 monoclonal antibodies or with peptides that compete with natural ligand RON2 for binding to the AMA1 (Lamarque et al., 2011; Tyler and Boothroyd, 2011). Both strategies prevent the assembly of a functional tight junction at the time of red cell invasion by merozoites.

However, incubation of invasive merozoites with molecular entities that compete for binding to the AMA1 groove such as RON2L peptide (Srinivasan et al., 2011), R1 peptide (Richard et al., 2010), or the potent small-molecules such as NCG00015280 and NCG00181034 (Srinivasan et al., 2013), as expected, disrupt the tight junction formation and inhibit the red cell invasion. However, despite a failure to form an intact tight junction, electron micrographs of invading merozoites reveal formation of evacuoles in the host cell in all these treatment conditions. Notably, anti AMA1 monoclonal antibodies that masked the binding groove of AMA1 block the assembly of the tight-junction as expected but also the formation of evacuoles (Srinivasan et al., 2011), suggesting that the AMA1 groove occupancy is necessary to signal rhoptry secretion.

On the other hand, evidence supporting the role of EBA-175-Glycophorin A as a trigger for rhoptry secretion in merozoites remains inconclusive. The original study relied on immunofluorescence and flow cytometry to monitor surface translocation of the rhoptry proteins CLAG3 and Rh2b in merozoites exposed to glycophorin A or RBC ghosts (Singh et al., 2010). However, since the study did not employ electron microscopy to observe evacuoles or changes in the rhoptry morphology upon incubation with ghosts, the evidence remains inconclusive. More recently, cryo-electron microscopy has been used to image rhoptries in the merozoites incubated with Glycophorin A (Martinez et al., 2022). No detectable changes in rhoptry volume or density were observed in the merozoites incubated with purified Glycophorin A (Martinez et al., 2022). Cryo-EM imaging data therefore does not support the EBA-175–glycophorin A interaction as a trigger for rhoptry secretion (Martinez et al., 2022). However, this study (Martinez et al., 2022) did not examine rhoptry secretion in the presence of peptides or small molecules that bind the AMA1 groove suggesting that more work is needed to conclusively demonstrate the role of these ligands in rhoptry secretion.

Recent studies have identified apicomplexan microneme proteins—CLAMP, SPATR, and CLIP—as essential factors for rhoptry secretion in *Toxoplasma* tachyzoites (Valleau et al., 2023). These proteins are also critical for blood-stage development in *Plasmodium*, as their conditional ablation results in severe defects in erythrocyte invasion (Valleau et al., 2023). However, their precise role in regulating rhoptry secretion in *Plasmodium* merozoites remains to be elucidated.

Several important studies have implicated the Rh5–CyRPA–Ripr complex—an essential assembly of secreted ligands—in mediating Ca²^+^ release during merozoite invasion of red blood cells (Weiss et al., 2015; Volz et al., 2016). These conclusions were primarily based on live-cell imaging of invasion events in red blood cells preloaded with the Ca²^+^-sensitive dye Fluo-4 AM. Conditional ablation of Rh5 or its binding partners, CyRPA or Ripr, abolished the transient Ca²^+^ signal observed in invaded red blood cells, leading to the proposal that the Rh5 complex may form the physical basis for this Ca²^+^ flux.

However, subsequent structural and functional analyses of the high–molecular weight Rh5–CyRPA–Ripr complex challenge this interpretation. While certain components of the complex can insert into the host membrane, the assembled complex itself does not measurably enhance Ca²^+^ permeability of the red blood cell membrane (Wong et al., 2019). Consistent with this, more recent structure–function studies demonstrated that locking Rh5 in a disulfide-constrained conformation does not impair its role in invasion, nor does it support a role in pore formation or Ca²^+^ release (Farrell et al., 2024). Together, these findings argue against the Rh5 complex functioning as a Ca²^+^-permeable pore.

Intriguingly, super-resolution imaging of invading merozoites suggests an alternative explanation for the observed Fluo-4 signal. The signal appears to originate at the rhoptry neck, raising the possibility that Fluo-4 diffuses from the red blood cell cytoplasm into the rhoptry through a transient pore or membrane discontinuity. In this model, Ca²^+^ would be released from the rhoptries into the host cell cytoplasm. Within this framework, it is plausible that the Rh5–CyRPA–Ripr complex functions not as a pore-forming unit, but rather as a signaling module that triggers the opening of this conduit between the merozoite rhoptries and the host red blood cell.

### 2.5 More C2 domains than meet the eye

Vesicle exocytosis requires membrane fusion, a process mediated by specialized fusion proteins. In *Plasmodium*, however, the machinery underlying microneme and rhoptry secretion appears to diverge from classical eukaryotic models, relying instead on atypical membrane fusion proteins. Intriguingly, many proteins implicated in apical organelle exocytosis still contain C2 domains. These ubiquitous eukaryotic modules, typically ~130 amino acids in length, adopt a compact β-sandwich structure composed of four-stranded β-sheets. The resulting core pocket can coordinate Ca²^+^ and mediate interactions with phospholipid membranes (Corbin et al., 2007). Notably, C2 domains are functionally diverse: while some are Ca²^+^-responsive, others are not, and they can also differ in lipid-binding specificity (Friedrich et al., 2010). This diversity positions C2 domains as versatile regulatory modules that can control membrane targeting and fusion in a context-dependent manner (Marty et al., 2013).

In *Plasmodium* merozoites, C2 domain–containing proteins are thought to play critical roles in membrane engagement and the regulated exocytosis of micronemes and rhoptries during host cell invasion. Several such proteins—including NdP2 (a component of the rhoptry secretory apparatus, RSA), Ferlin2, PfRASP2 or PfCERLI1—harbor C2 domains; notably, RASP2/CERLI1 also contain Pleckstrin homology (PH) domains. These proteins localize to distinct loci on the rhoptries and appear to regulate secretion in a spatially organized and multi-step manner (Suarez et al., 2019; Coleman et al., 2018; Sparvoli and Lebrun, 2021; Martinez et al., 2022).

RASP2 localizes to the rhoptry cap and is proposed to mediate fusion of the rhoptry with the apical vesicle, whereas NdP2 and Ferlin2 localize at the apical vesicle–RSA interface and are likely required for AV–RSA fusion (**Figure 2**). This spatial segregation suggests that rhoptry secretion is a coordinated, stepwise process governed by distinct molecular components. The enrichment of C2 domains among these essential proteins raises important questions regarding the role of calcium in this process. Specifically, is rhoptry secretion Ca²^+^-responsive? More broadly, could the temporal and spatial regulation of microneme and rhoptry secretion be encoded by variation in the calcium sensitivity and lipid-binding properties of their C2 domain mediators?

C2 domain, as expected is also present in proteins that mediate microneme secretion. DOC2.1 protein is necessary for microneme exocytosis, translating the rise in intracellular Ca^+2^ into a vesicle fusion event, at the time of merozoite invasion in *Plasmodium* (Farrell et al., 2012). Apart from the calcium responsiveness, the C2 domain containing proteins confer lipid selectivity (Corbin et al., 2007).

An essential lipid-binding protein PfRASP2 (Rhoptry Apical Surface Protein) (Suarez et al., 2019) or PfCERLI1 (Cytosolically Exposed Rhoptry leaflet Interacting protein) (Liffner et al., 2020), has been shown to localize to the cytoplasmic face of the rhoptry organelle membrane, is essential for rhoptry exocytosis and invasion, contain both C2 and Plekstrin homology domains required for lipid binding (Suarez et al., 2019; Liffner et al., 2020). *In vitro* biochemical assays have validated the lipid-binding nature of RASP2 or CERLI1 (Suarez et al., 2019). Likewise, Ferlin2, a member of the Nd complex in tachyzoites, refractory to gene knockout in both *Toxoplasma* and *Plasmodium* has C2 domains that presumably acts as a calcium sensor for rhoptry secretion perhaps at the AV-RSA fusion step. Ferlin proteins can act as Ca²^+^-dependent triggers for membrane fusion, variable C2 domain architecture within can encode complex calcium responsiveness (Marty et al., 2013). In tachyzoites, Ferlin2 is essential for rhoptry exocytosis and formation of the moving junction (Coleman et al., 2018). However, Ferlin2 has not yet been characterized in *Plasmodium*. Consistent with their role in regulating rhoptry secretion, these genes are expressed late in schizogony, and their ablation severely impairs merozoite invasion.

### 2.6 Old questions and new hypotheses

In the current model of merozoite invasion, several rhoptry-resident proteins that are essential for host cell entry—such as Rh5 and the RON complex—are secreted prior to complete rhoptry discharge. Rhoptries, which exhibit a characteristic club-shaped morphology, have been proposed to be compartmentalized into two regions: a slender, duct-like apical segment termed the neck, and a lipid-rich, globular basal region referred to as the bulb (Roger et al., 1988; Ghosh et al., 2017; Counihan et al., 2013). These regions are thought to possess distinct protein compositions, with Rh and RON proteins predominantly localized to the rhoptry neck. However, it has remained unclear whether this apparent compartmentalization reflects a true physiological basis for the temporally regulated and sequential release of rhoptry contents, or merely a structural organization. Strikingly, recent *in situ* cryo-electron tomography of vitrified merozoites has revealed that rhoptries display a largely homogeneous, granular internal architecture, with no obvious evidence of discrete subcompartments (Sun et al., 2024). This observation challenges the conventional compartmentalization model and raises important questions about the mechanisms that enable the ordered secretion of rhoptry neck proteins while preventing premature release of bulb contents.

Intriguingly, a recent cryo-electron tomography study of developing merozoites within schizonts revealed images of the apical complex in which the apical vesicle (AV) was clearly positioned at the rhoptry tips (Bisson et al., 2021). However, this structure was not recognized as a distinct entity and was instead annotated as part of the rhoptry. Subsequent studies have since established the definitive existence of the AV and begun to elucidate the molecular machinery regulating its fusion with the rhoptry secretion apparatus (RSA) and rhoptries (Mageswaran et al., 2021; Martinez et al., 2022). Together, these observations raise the possibility that invasion-critical proteins such as Rh5 and the RON complex may localize to the apical vesicle (AV) rather than the rhoptry neck, and are secreted from this intermediate compartment, potentially in coordination with microneme discharge. The formation of the tight junction during merozoite invasion requires the coordinated secretion of RON proteins (traditionally assigned to the rhoptry neck) and AMA1, a microneme protein, suggesting the existence of a tightly coupled secretory mechanism.

This model is further supported by the localization of calcium-dependent membrane fusion proteins, such as Ferlin2, at the interface between the RSA and the AV, as demonstrated in tachyzoites (Aquilini et al., 2021; Sparvoli and Lebrun, 2021). Importantly, disruption of Ferlin2 function in tachyzoites prevents moving junction assembly (Coleman et al., 2018), providing functional evidence for the role of this interface in regulated secretion. Moreover, the presence of distinct molecular machinery governing AV–RSA fusion and rhoptry–AV fusion is consistent with a model of sequential secretion, in which rhoptry neck and bulb contents are released in a stepwise and regulated manner during host cell invasion. Nevertheless, these propositions remain speculative and should be considered testable hypotheses, requiring direct experimental validation to establish their physiological relevance.

Taken together, these findings support a model in which rhoptry secretion depends on prior microneme secretion. In this framework, microneme proteins such as AMA1 and the CLAMP complex, upon translocation to the host cell surface, may sense productive host cell attachment and transmit this information through as-yet-unidentified signaling pathways. These signals are proposed to activate lipid-binding proteins that regulate rhoptry–AV or AV–RSA membrane fusion by responding to localized changes in lipid composition or free Ca^+2^ near the merozoite plasma membrane, which may occur following host cell egress or during microneme discharge, thereby licensing rhoptry secretion.

## 3 Physiology of gliding motility

Over a century ago, pioneering malariologists observed that malaria parasites are motile (Mccallum, 1898; Ross, 1899). They noted that the extracellular invasive stages—sporozoites and ookinetes—move using a distinctive mechanism that does not involve cilia, flagella, or the shape changes typical of amoeboid motility. Pioneering studies on the motility of *Plasmodium* sporozoites exhibited complex helical motion on the glass surface. Motile sporozoites left behind traces of secreted antigens, such as the Circumsporozoite protein, as they ‘glided’ along it (Stewart and Vanderberg, 1991). This peculiar form of motility was termed gliding motility. Early video microscopy studies of viable sporozoites revealed movement of the latex beads, secreted proteins, and protein-antibody complexes on the cell surface along the long axis from anterior to posterior end, resulting in accumulation of particles at the posterior end of the cell, a process referred to as ‘capping’ (King, 1988). The distinctive, conveyor-belt-like movement of particles on the zoite surface suggested an active motor system beneath the membrane, providing early clues to the mechanistic basis of gliding motility. Later, this motility was explained by a linear motor model (Soldati et al., 2004). This peculiar form of motility is also found in the invasive stages of other apicomplexan parasites and is necessary for their virulence and dissemination (Sibley et al., 1998).

Early studies on red blood cell invasion showed that actin polymerization inhibitors, such as Cytochalasin-D, block merozoite invasion, suggesting that invasion requires actin-myosin-based motility (Miller et al., 1979; Pinder et al., 1998). Despite this, the structural evidence for actin filaments remained elusive in *Plasmodium* or *Toxoplasma* for a long time. However, a well-designed experimental approach using cytochalasin D-resistant parasites and host cells demonstrated that parasite actin is required for tachyzoite invasion of host cells (Dobrowolski and Sibley, 1996). For a long time, it was believed that *Plasmodium* actin forms highly unstable and unusual short filaments, about 100 nm in length, and that this long-eluded characterization by conventional biochemical or imaging techniques (Schmitz et al., 2005; Schüler and Matuschewski, 2006). High-resolution, low-voltage field-emission scanning electron microscopy of parasites treated with actin stabilizers revealed that actin filaments are primarily confined to a tight-geometry compartment beneath the plasma membrane in tachyzoites (Schatten et al., 2003). The discovery of an unconventional myosin motor and its localization right beneath the sporozoite plasma membrane in this compartment finally put the crucial piece in the puzzle (Bergman et al., 2003).

The motility of *Plasmodium* zoites is thus driven by the actin-myosin motor encased in the cortical space. The force generated by the actin myosin motor must be translated into motility. It was therefore proposed that surface molecules known as adhesins, which facilitate the adhesion of zoites to the surface and to host cells, must somehow engage with the actin filaments that tread on the myosin heads (Tardieux and Baum, 2016). The adhesins seem to move rearwards from the anterior pole of the zoite surface, reminiscent of the capping reaction. These observations led to widespread acceptance of a unidirectional linear motor model for the motility of *Plasmodium* zoites (Soldati et al., 2004).

**Figure 3:**
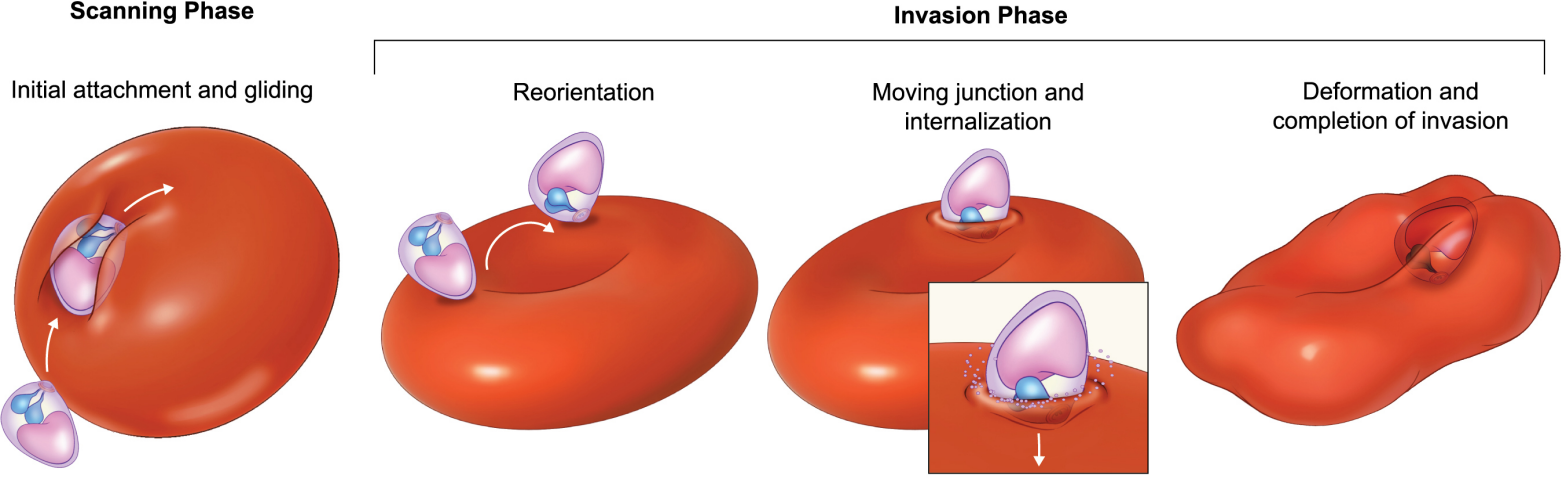
The revised scheme of *Plasmodium* merozoite invasion of red blood cell. Initial contact of the merozoite with the red blood cell (RBC) induces surface gliding and membrane deformation. Gliding motility is a conserved prerequisite for host cell engagement, and the degree of membrane deformation correlates with invasion success, serving as a readout of host cell suitability during the pre-invasion scanning phase. The invasion phase proceeds through merozoite reorientation, formation of the moving junction, and internalization into the RBC.

Merozoite invasion of red blood cells is an active process driven by the formation of the moving junction, a receptor–ligand complex that translocate rearward along the parasite surface via the actin–myosin motor (**Figure 3**) (Aikawa et al., 1978). Disruption of either actin or myosin abrogates invasion, underscoring the central role of this motor in host cell entry. Despite this, merozoites were long considered incapable of gliding motility (Baum et al., 2006), an assumption that persisted for decades following early videographic analyses of invasion (Dvorak et al., 1975). This view has been fundamentally revised by the recent research (Weiss et al., 2015; Yahata et al., 2021). Most important experimental evidence was provided by the work of Yahata et al. (2021), who demonstrated that *Plasmodium* merozoites do exhibit gliding motility when allowed to move on an appropriate hydrophilic substrate. Strikingly, their findings further revealed that the broader end of the merozoite functions as the apical pole, overturning the long-standing model of merozoite polarity (**Figure 1**). Together, these observations necessitate a re-evaluation of the mechanistic framework of invasion, particularly with respect to its kinetics, force generation, and spatial organization.

### 3.1 The conserved cellular organization of the motor

A defining cellular feature of alveolates is the presence of flattened membranous sacs, termed alveoli, positioned immediately beneath the plasma membrane. These structures are highly specialized in both architecture and function, enabling diverse alveolate lineages to adapt to a wide range of ecological niches and parasitic lifestyles. Consequently, alveoli support distinct physiological roles across species and contribute significantly to the diversity of cellular organization within the Alveolata (Kono et al., 2013; Ferreira et al., 2021).

In apicomplexan zoites, alveoli are further specialized to form the Inner Membrane Complex (IMC), a double-membrane system underlying the plasma membrane (Kono et al., 2013). The IMC can be conceptualized as a quasi-continuous capsular structure that provides mechanical support, serves as a scaffold for the cytoskeleton, and anchors the molecular machinery responsible for gliding motility—the glideosome. Structurally, the IMC consists of closely apposed inner and outer membranes that are fused at the apical and basal ends. In *Plasmodium* merozoites, sporozoites, and ookinetes, the IMC exhibits a conserved organization, typically comprising a single vesicular unit associated with a network of subpellicular microtubules (Kono et al., 2013). Functionally, the two IMC membranes are asymmetrically specialized. The inner membrane, oriented toward the cytoplasm, harbors a complex array of proteins that mediate interactions with cytoskeletal elements and microtubules. In contrast, the outer membrane, facing the plasma membrane, anchors components of the glideosome. The narrow space between the IMC and the plasma membrane—referred to as the cortical or pellicular space—forms a dedicated compartment that houses the actin–myosin motor and associated factors required for motility (Ferreira et al., 2021).

In *Plasmodium falciparum* merozoites, there are only 3–5 microtubules, located beneath the cytoplasmic membrane of the IMC, and all microtubules are on the same side of the cell (Hanssen et al., 2013; Sun et al., 2024; Ferreira et al., 2021) (**Figure 1**). High-resolution cryo-ET data provide evidence for the discontinuous nature of the IMC. The double-membrane IMC is discontinuous at the apical and basal ends of the zoites; the two IMC membranes are sealed at the ends (**Figure 1**) (Hanssen et al., 2013; Sun et al., 2024). The apical end forms an apical collar that accommodates apical rings and apical secretory organelles in *Plasmodium* sporozoites and merozoites (Sun et al., 2024). The IMC in merozoites appears to have fenestrations at other loci as well (**Figure 1**) (Hanssen et al., 2013). These fenestrations can facilitate biochemical coupling between the cortical space and the cytoplasm, enabling metabolite exchange and the trafficking of molecular components required for glideosome assembly and function.

Emerging evidence from closely related apicomplexan zoites indicates that actin filaments stream through the pellicular space in a highly regulated manner (Martinez et al., 2022; Hvorecny et al., 2024). Actin nucleation is thought to occur at the apical end of the zoite, after which filaments are directed into the narrow pellicular (cortical) space, where they undergo continuous rearward flux. This actin flux is essential for gliding motility, providing a dynamic substrate for force generation (Hvorecny et al., 2024). Mechanistically, filamentous actin interacts with myosin motors anchored to the outer membrane of the IMC, thereby generating the traction forces required for parasite movement.

Although such studies are currently lacking in *Plasmodium* merozoites, insights from sporozoites suggest that the confined geometry of the pellicular space plays a critical role in regulating actin dynamics. Notably, this spatial constraint enables the local concentration of F-actin to reach extraordinarily high levels—estimated to be on the order of ~60 mM near the basal end. This represents a concentration approximately two orders of magnitude higher than that observed in most eukaryotic cells, or even the global F-actin levels within the sporozoite (Pražák et al., 2025). These studies demonstrate a dynamic exchange of actin filaments through basal IMC fenestrations in sporozoites, and further suggest that the apical and basal poles likely serve as key regulatory sites for pellicular actin organization (Hvorecny et al., 2024; Pražák et al., 2025), a principle that may also extend to merozoites. Collectively, these findings challenge long-standing assumptions regarding the nature of *Plasmodium* actin, which was previously considered to form highly unstable, exceptionally short filaments of approximately ~100 nm in length (Schmitz et al., 2005; Schüler and Matuschewski, 2006).

### 3.2 Gliding motility in merozoites

Gliding motility has been intensively studied for *Toxoplasma gondii* tachyzoites and *Plasmodium berghei* sporozoites; in both cases, the motility is associated with a helical motion in three-dimensional matrices. The helical motion associated with gliding motility can also be observed during host cell entry. Gliding motility is characterized by a helical, or corkscrew, motion of the zoites (Lettermann et al., 2024).

Gliding motility observed in *Plasmodium* merozoites is bona fide; merozoites released post egress were found to exhibit an associated corkscrew motion of the cell, which wasn’t discernible in the *falciparum* merozoites due to their small size, but was readily seen in larger *knowlesi* merozoites (Yahata et al., 2021). Merozoite motility was also substrate-dependent. Merozoites favored hydrophilic surfaces but had little motility on the BSA-coated glass slides (a suitable substrate for observing sporozoite motility) (Yahata et al., 2021). Knowlesi merozoites appear to exhibit enhanced gliding motility on poly-L-lysine-coated surfaces. Inducible gene ablation of essential glideosome components, such as Actin 1 (ACT1), the predominant actin expressed during the blood stages, or GAP45 or Glideosome Associated Protein 45, impaired gliding motility in merozoites, consistent with their conserved roles in gliding motility (Yahata et al., 2021). Although a high-resolution Cryo-ET characterization of the merozoite motility motor and cellular actin organization is still lacking, the conserved glideosome components and IMC organization in sporozoites and merozoites indicate that these stages share an equivalent underlying structural and functional framework. Sporozoites and merozoites are both capable of gliding motility, albeit they have significant differences in their gliding speeds (see **Table 1**) and the duration of motility consistent with their biological roles.

**Table 1:** Gliding motility speed and duration for different zoites. Note the variability in motility speeds and durations.

Species	Stage	Gliding speed	Duration of gliding
*Plasmodium falciparum*	Merozoite	0.6 μm/s	43 seconds
*Plasmodium knowlesi*	Merozoite	1.1 μm/s	316 seconds
*Toxoplasma gondi*	Tachyzoite	2.6 μm/s	600 seconds
*Plasmodium berghei*	ookinete	.096 μm/s	–
*Babesia bovis*	Merozoite	6 μm/s	125 seconds
*Plamsodium yoelli*	Sporozoite	5 μm/s	600 seconds
*Plamsodium berhgei*	Sporozoite	2 μm/s	–

The modulation of intracellular Ca^+2^ in the merozoite by the use of calcium ionophore did not significantly affect the gliding motility of the merozoites; however, chelation of intracellular Ca^+2^ by BAPTA-AM impaired gliding motility (Yahata et al., 2021). These findings indicate that, unlike microneme secretion, gliding motility is not triggered by a spike in intracellular Ca^+2^. However, a basal level of intracellular Ca^+2^ appears to be required for merozoites to exhibit motility, likely a physiological minimum of Ca^+2^ ions needed for the secretion of adhesins from micronemes and for the activity of calcium-dependent protein kinases (CDPKs), which are necessary for motility (Kato et al., 2008). In their study (Yahata et al., 2021) tested a set of chemical modulators to probe signaling pathways that regulate gliding motility in *Plasmodium falciparum* merozoites. In their assays, they reported that the PI-PLC (phosphoinositide-specific phospholipase C) inhibitor **⁠**U73122 inhibited gliding motility, whereas its inactive analog U73343 did not. They also showed that the DAG kinase inhibitor R59022 affected motility, but that neither propranolol nor general DAG inhibitors had an effect on merozoite gliding. These observations are intriguing and suggest a possible role for merozoite signaling pathways in gliding motility. Prior studies have implicated guanosine 3′,5′-cyclic monophosphate (cGMP) signaling and the activity of the cyclic nucleotide-degrading phosphodiesterase PDEδ in driving gliding motility in *Plasmodium* ookinetes (Moon et al., 2009). However, a comparable mechanism linking (cGMP) signaling to gliding motility in merozoites has not yet been established conclusively.

The demonstration of gliding motility in *Plasmodium* merozoites has helped explain an essential facet of red cell invasion, the pre-invasion deformation of the red blood cell by the merozoite (Weiss et al., 2015; Yahata et al., 2021). Pre-invasion red blood cell deformation is strongly correlated with successful invasion (Weiss et al., 2015; Blake et al., 2020). It may be involved in host cell recognition and sensing, or in priming the merozoite for downstream invasion events. This phase may therefore be referred to as the scanning phase (**Figure 3**). The merozoite’s ability to deform the red blood cell depends on gliding motility; chemical inhibition of either the actin polymerization by Cytochalasin D or inducible genetic ablation of the essential genes of the motor, such as ACT1 or GAP45, impairs the ability of merozoites to deform or invade the red blood cells (Yahata et al., 2021). Studies using genetically modified parasites lacking EBA175, or selectively expressing EBA175, suggest that EBA/PfRh protein interactions predominantly drive strong red blood cell deformation during pre-invasion (Weiss et al., 2015).

Importantly, it was found that inducible knockdown of the AMA-1 gene (a key component of the moving junction) or the chemical disruption of the AMA1-RON2 interaction did not affect the merozoites’ ability to deform red blood cells, but severely impaired their ability to invade red blood cells (Weiss et al., 2015), indicating that the gliding motility of merozoites depends on different molecular interactions for scanning/weak deformation and invasion. The molecular interaction between AMA-1 and RON2 commits the merozoite to the invasion (Riglar et al., 2011, Srinivasan et al., 2011).

### 3.3 Moving frontiers and new questions

An important question is how the actin-myosin motor caters to the different forms of motility required during invasion. During the scanning phase, the merozoite glides along the red blood cell surface, a step necessary for pre-invasion deformation. Heparin treatment severely compromises the pre-invasion deformation by merozoites, suggesting that this step requires yet-undiscovered adhesins. Strong deformation necessitates motor engagement with EBA/Rh ligands. However, during invasion or internalization, the motility motor of the merozoite must facilitate the internalization through the ‘moving junction’. The motor activity must therefore shift from an initial low-force exploratory state to a high-force contractile state during internalization. Indeed, biochemical and structural data suggest that motor activity is tunable in invasive merozoites; the motor can be switched from a low-force scanning mode to a high-force mode observed during internalization (Robert-Paganin et al., 2019). This is achieved by regulating the activity of PfMyoA, the main myosin motor responsible for force generation, through phosphorylation of a key residue that controls the motor’s duty ratio. Phosphorylation of PfMyoA changes how the motor functions. The rigor-like structure shows that phosphorylation of S19 in the N-terminal extension allows it to interact with the converter domain during the final step of the lever-arm movement, which occurs when ADP is released (Robert-Paganin et al., 2019). This interaction determines how long PfMyoA remains strongly attached to actin. By altering this attachment time, phosphorylation adjusts the motor’s speed and the force it generates. As a result, a single phosphorylation event alters the duty ratio of class XIV myosins, enabling the motor to switch between generating force under load during host cell invasion and moving actin rapidly under low-load conditions, such as during exploratory gliding. During the internalization phase of merozoite invasion of the red cell, the motor undergoes dephosphorylation to produce a higher force under loaded conditions (Robert-Paganin et al., 2019).

It remains unclear how, or even whether, the motility motor can dynamically engage, disengage, and re-engage with different ligands or adhesion complexes to facilitate these rapid, successive steps during red blood cell invasion. A mechanism involving the turnover of adhesion sites has been demonstrated to explain sporozoite motility, in which TRAP primarily mediates the formation and rupture of adhesion sites, and the turnover of discrete adhesion sites modulates sporozoite motility (Münter et al., 2009; Gilson, 2009). In addition to the role of actin dynamics, proteolytic shedding of surface adhesins or merozoite surface proteins may also serve as a mechanism to disengage previously engaged adhesins, thereby allowing productive force transmission through the engagement of newly recruited adhesins with the actomyosin motor. It is intriguing to speculate that such mechanisms may also operate in merozoites during the pre-invasion scanning and internalization phase (**Figure 3**).

Because the mechanism of gliding motility is highly conserved between *Plasmodium* and *Toxoplasma* and shares a broadly similar structural and functional framework, insights from tachyzoite motility studies provide an important foundation for understanding merozoite motility. In this context, a recent model proposes that actin dynamics in the subpellicular space can be described as an emergent flow phenomenon underlying distinct modes of gliding motility (Hueschen et al., 2024). This framework, grounded in continuum theory, treats actin as a collective system whose self-organized states give rise to the different motility behaviors observed experimentally. Within this model, heterogeneous or transient patterns of actin distribution in the pellicular space, including meandering patches and directional filament flow, are interpreted as manifestations of distinct self-organized actin states. While conceptually appealing and useful in rationalizing several imaging observations, the model remains largely theoretical and lacks substantial direct experimental validation. A key unresolved question is whether these putative actin states are mechanistically coupled to adhesin dynamics, which are required for effective transmission of force generated by the actomyosin motor.

In particular, the efficiency and nature of force transmission depend critically on the molecular “clutch” linking the actomyosin system to surface adhesins. Variations in gliding behavior may therefore arise from differences in which adhesins are engaged at a given time, since adhesin identity and distribution determine how mechanical force is coupled across the parasite surface. Polarized secretion and spatial gradients of adhesins on the zoite surface have been reported (Gantt et al., 2000; Kappe et al., 1999), providing a potential basis for directional bias and variability in motility modes observed across apicomplexan stages, including tachyzoites and *Plasmodium* merozoites.

Notably, the effective flow rate of actin through the pellicular space—which depends on factors such as actin polymerization and depolymerization kinetics, compartment geometry, and the density and organization of myosin motors in the IMC—may also contribute to the broad spectrum of motility speeds observed among apicomplexan zoites (**Table 1**). Additional regulatory layers, not yet fully defined, are likely to further modulate this system.

## 4 Conclusions

Large-scale functional profiling of the *Plasmodium* genome shows that ~63% of genes are required for asexual or blood stage growth—an unparalleled degree of genetic requirement among unicellular eukaryotes that reflects the extreme evolutionary specialization of the asexual blood stage (Bushell et al., 2017). The identification of SIP2 as an essential transcription factor for merozoite formation further exposes the depth and coordination of the gene regulatory programs governing merozoite differentiation (Nishi et al., 2025). Despite this complexity, little progress has been made in understanding the physiology of the invasive merozoites.

Recent advances in *Plasmodium* experimental genetics, improved methods for merozoite isolation, and the convergence of biochemical and high-resolution imaging approaches have enabled parasitologists to gain deeper insights into merozoite biology. Large-scale mutagenesis screens (Zhang et al., 2018) and stage-specific transcriptomic analyses across *Plasmodium* species informs on the genes essential for merozoite invasion. However, the highly divergent gene usage patterns and extreme AT-richness of the *Plasmodium* genome continue to limit the reliability of computational predictions of gene function, necessitating careful experimental validation. *Plasmodium* merozoites have diverged substantially, shaped by the unique evolutionary pressures of rapid red blood cell invasion and blood-stage proliferation. Dissecting merozoite physiology remains challenging due to their minute size, brief viability and high fragility outside the host cell. As a result, progress in merozoite biology remains constrained by slow, meticulous, reductionist approaches requiring great technical expertise and effort.

Unprecedented resolution afforded by modern cryo-electron microscopy has enabled detailed visualization of merozoite subcellular structures, leading to the identification of the rhoptry secretion complex, the apical vesicle, and distinct classes of apical secretory organelles (Martinez et al., 2022; Theveny et al., 2022; Sun et al., 2024; Anton et al., 2022). Of particular note is the discovery of the rhoptry secretion apparatus (RSA) in *Plasmodium* merozoites, which has opened a new frontier for dissecting both conserved and repurposed features of alveolate biology in the context of merozoite invasion (Mageswaran et al., 2021; Martinez et al., 2022). However, despite the identification of conserved proteins involved in apical organelle secretion, their integration into the second-messenger signaling pathways operating in invasive merozoites remains to be established.

Invasive merozoites perform complex maneuvers such as motility, host cell recognition, apical organelle secretion and motility driven internalization likely regulated by extensive signaling pathways. Notably, recent advances have substantially deepened our understanding of how Ca^+2^ and cyclic mononucleotide signaling intersect during merozoite invasion (Balestra et al., 2021; Kuehnel et al., 2023). But further studies are needed to understand how merozoites sense cues such as changes in ionic milieu experienced during host cell egress and mechanical contact with the host red blood cell during invasion. The discovery of the bona fide gliding motility in *Plasmodium* merozoites has been foundational, as it led to the redefinition of not only the merozoite invasion physiology but also its morphology (Yahata et al., 2021). However, the regulation of motility and its integration with Ca^+2^ or cGMP or cAMP signaling is still awaited.

Advances in imaging, the increasing ease of genetic manipulation, and improved methods for harvesting merozoites are poised to usher malaria parasitology and cell biology into a new era focused on the physiology of the merozoite. Insights from these studies may drive the development of novel antimalarial strategies and strengthen humanity’s fight against this ancient disease.
